# Cooperative Strategies to Develop Effective Stroke and Heart Attack Awareness Messages in Rural American Indian Communities, 2009–2010

**DOI:** 10.5888/pcd10.120277

**Published:** 2013-05-16

**Authors:** Carrie S. Oser, Dorothy Gohdes, Crystelle C. Fogle, Fawn Tadios, Velva Doore, Doreen S. Bell, Todd S. Harwell, Steven D. Helgerson

**Affiliations:** Author Affiliations: Dorothy Gohdes, Crystelle C. Fogle, Todd S. Harwell, Steven D. Helgerson, Montana Department of Public Health and Human Services, Cardiovascular Health Program, Helena, Montana; Fawn Tadios, Chippewa Cree Tribal Health, Box Elder, Montana; Velva Doore, Doreen S. Bell, Fort Belknap Tribal Health Department, Harlem, Montana.

## Abstract

**Introduction:**

National initiatives to improve the recognition of heart attack and stroke warning signs have encouraged symptomatic people to seek early treatment, but few have shown significant effects in rural American Indian (AI) communities.

**Methods:**

During 2009 and 2010, the Montana Cardiovascular Health Program, in collaboration with 2 tribal health departments, developed and conducted culturally specific public awareness campaigns for signs and symptoms of heart attack and stroke via local media. Telephone surveys were conducted before and after each campaign to evaluate the effectiveness of the campaigns.

**Results:**

Knowledge of 3 or more heart attack warning signs and symptoms increased significantly on 1 reservation from 35% at baseline to 47% postcampaign. On the second reservation, recognition of 2 or more stroke signs and symptoms increased from 62% at baseline to 75% postcampaign, and the level of awareness remained at 73% approximately 4 months after the high-intensity campaign advertisements ended. Intent to call 9-1-1 did not increase in the heart attack campaign but did improve in the stroke campaign for specific symptoms. Recall of media campaigns on both reservations increased significantly from baseline to postcampaign for both media outlets (ie, radio and newspaper).

**Conclusion:**

Carefully designed, culturally specific campaigns may help eliminate disparities in the recognition of heart attack and stroke warning signs in AI communities.

## MEDSCAPE CME

Medscape, LLC is pleased to provide online continuing medical education (CME) for this journal article, allowing clinicians the opportunity to earn CME credit.

This activity has been planned and implemented in accordance with the Essential Areas and policies of the Accreditation Council for Continuing Medical Education through the joint sponsorship of Medscape, LLC and Preventing Chronic Disease. Medscape, LLC is accredited by the ACCME to provide continuing medical education for physicians.

Medscape, LLC designates this Journal-based CME activity for a maximum of 1 **AMA PRA Category 1 Credit(s)™**. Physicians should claim only the credit commensurate with the extent of their participation in the activity.

All other clinicians completing this activity will be issued a certificate of participation. To participate in this journal CME activity: (1) review the learning objectives and author disclosures; (2) study the education content; (3) take the post-test with a 70% minimum passing score and complete the evaluation at www.medscape.org/journal/pcd (4) view/print certificate.

**Release date: May 15, 2013; Expiration date: May 15, 2014**

### Learning Objectives

Upon completion of this activity, participants will be able to:

- Report the current trend regarding heart disease and stroke mortality in AIs
- Describe public education campaign strategies that may be utilized in educating AI communities
- Report the effects of heart attack and stroke-related public awareness campaigns within an AI community
- Assess the results in the need to seek treatment after public education campaigns regarding heart attack and stroke were incorporated in AI communities

**EDITORS**

Rosemarie Perrin, Editor, Camille Martin, Editor; *Preventing Chronic Disease*. Disclosure: Rosemarie Perrin and Camille Martin have disclosed no relevant financial relationships.

**CME AUTHOR**

Charles Vega, MD, Associate Professor and Residency Director, Department of Family Medicine, University of California-Irvine, Irvine. Disclosure: Charles P. Vega, MD, has disclosed no relevant financial relationships.

**AUTHORS AND CREDENTIALS**

Disclosures: Carrie S. Oser, MPH; Dorothy Gohdes, MD; Crystelle C. Fogle, MBA, MS, RD; Fawn Tadios; Velva Doore; Doreen S. Bell, MHA; Todd S. Harwell, MPH; and Steven D. Helgerson, MD, MPH, have disclosed no relevant financial relationships.

Affiliations: Carrie S. Oser, Dorothy Gohdes, Crystelle C. Fogle, Todd S. Harwell, and Steven D. Helgerson, Montana Department of Public Health and Human Services, Cardiovascular Health Program, Helena, Montana; Fawn Tadios, Chippewa Cree Tribal Health, Box Elder, Montana; Velva Doore, Doreen S. Bell, Fort Belknap Tribal Health Department, Harlem, Montana.

## Introduction

Cardiovascular disease was notably rare in several American Indian (AI) tribes for many years but has now become a leading cause of death (1). The Strong Heart Study documented high rates of coronary heart disease and stroke in AIs in several regions of the United States (2–4). A national analysis of heart disease and stroke mortality in AIs and Alaska natives showed that heart disease death rates, which had been 21% lower than rates for the total US population in the 1970s, were 20% higher than US rates by the late 1990s; stroke death rates became 14% higher among AIs than among US adults over the same period (5). Disparities in adult awareness of heart attack warning signs and symptoms have become widely recognized (6,7). Although studies have shown that most AIs are aware of their risk for heart disease and stroke (8,9), few studies have assessed AIs knowledge of signs and symptoms. A recent study among urban Indian communities in the southwest United States showed that awareness of heart attack and stroke signs and symptoms was lower than that of the general US population (10). However, no systematic studies have been conducted in AI communities about their awareness of heart attack or stroke warning signs and the need to seek emergent treatment, key factors for survival of heart attack and stroke (11).

Stroke and heart disease death rates between 1996 and 2000 were approximately one-third higher among AIs in Montana than among whites, and these disparities have persisted in recent years (12,13). Because of the noted disparities in cardiovascular mortality, the Montana Department of Public Health and Human Services (DPHHS) Cardiovascular Health (CVH) Program recognized the need for culturally appropriate education concerning early warning signs of heart attack and stroke. AIs in Montana live in isolated rural areas where access to local television and billboards is limited, prohibitively expensive, or both. Each tribe has culturally distinct traditions, so developing and evaluating each campaign individually in cooperation with the local tribal health departments was essential. We report data from the evaluations of 2 campaigns and provide information about key processes that were used successfully to reach residents of rural Indian communities in Montana.

## Methods

The Montana DPHHS approached Rocky Boy’s and Fort Belknap tribal health departments individually to invite them to collaborate in the development and implementation of public education campaigns that target AI adults living on the reservations in north central Montana. The Rocky Boy’s heart attack campaign was conducted October 2009 to May 2010. The Fort Belknap stroke campaign also ran October 2009 to May 2010 and was followed by a maintenance campaign to enhance message retention. During the maintenance campaign, advertisements ran for 1 week every 3 weeks during a 16-week period from July 2010 through October 2010. Institutional review board approval was not required by the Montana DPHHS because previous research has established the effectiveness and safety of heart attack and stroke-related public awareness campaigns, and only de-identified data were used for analysis.

### Settings

Two tribal health departments in north central Montana worked with the Montana CVH Program to reach their tribal members. The Fort Belknap reservation encompasses more than 1,000 square miles; it has a population of 2,851, of which 95% is AI, and a median age of 25.8 years (14). The Rocky Boy’s reservation is Montana’s smallest reservation in terms of land mass, with 171 square miles; it has a population of 3,323, of which 97% is AI, and a median age of 22.5 years (14).

### Development of campaign messages

The essential first step in the development of the campaigns was to approach tribal health authorities and define the roles of tribal health and state health departments during the campaigns. A key role of the respective tribal health departments was to help convey each tribe’s unique cultural norm so that campaign approaches could be culturally appropriate. The Montana CVH Program contracted with a Montana communications firm to assist in the development and implementation of the campaigns. Focus groups were held on each reservation to obtain feedback from AI community members about sample themes of culturally appropriate advertisements and to identify community-specific barriers to calling 9–1-1. Community members were recruited with assistance of employees of the respective tribal health department and included men and women of varied ages in the target audience (AI adults aged 30 or older), diverse educational and work backgrounds, and a mixture of health and lifestyle traits. The focus groups — 1 for Fort Belknap (n = 16) and 1 for Rocky Boy’s (n = 25) — rated the importance of several preformatted statements about life-saving knowledge related to stroke or heart attack. Focus group participants reviewed and provided feedback on print advertisements and sample messages for Montana AIs. On the basis of input from focus group participants, print materials were developed to feature local residents, topography from each reservation, or both. Radio and theater advertisements featured AIs, and theater advertisements featured Montana topography.

### Public education campaign

Each high-intensity campaign was conducted during a 20-week period separated by a 10-week blackout period. On the basis of focus group input, the Rocky Boy’s overall campaign theme was “Protect your heart. Preserve our nation” and was incorporated in all print materials. The Rocky Boy’s media channels for the heart attack campaign included paid print, radio, and theater advertisements; print inserts, direct mailers, press releases, and a series of road signs were also used. Advertisements were featured on YouTube. To customize the campaigns further, local heart attack or stroke survivors were featured in press releases and newsletters that were sent as direct mailers. Similar media channels were used in the Fort Belknap stroke campaign. To aid message retention for the Fort Belknap stroke campaign, a maintenance campaign followed the high-intensity campaign with intermittent airing of advertisements over a 16-week period. For both campaigns, tribal health personnel were integral partners. They distributed customized posters and brochures and print inserts to various community organizations and businesses, including pharmacies, clinics, and senior centers. Tribal health directors approved all new materials, and their staff assisted with arranging the focus groups and conducting outreach during the campaigns.

### Evaluation

To evaluate the effect of the campaigns among AI adults aged 30 years or older living on the reservations, the Montana CVH Program conducted a systematic, random-sample telephone survey, at baseline (before any media activities began) and at postcampaign (immediately following the high-intensity campaign), for both the heart attack and the stroke campaigns; for the stroke campaign, the survey was also conducted postmaintenance (immediately following the maintenance campaign). Before conducting the survey, a screening process verified that the potential respondent was a reservation resident and asked whether a household member aged at least 30 years who was an AI was present to participate. Trained interviewers used computer-assisted telephone interviewing software to conduct the survey. When more than 1 potential respondent lived in a contacted household, 1 eligible respondent was randomly selected. Up to 15 attempts were made to complete unanswered calls. Respondents in all surveys were asked a series of questions from the Behavioral Risk Factor Surveillance System survey regarding sociodemographic information (age, sex, and years of education) and personal history of heart attack, angina, coronary heart disease, stroke, coronary artery bypass graft, angioplasty, diabetes, high blood pressure, high cholesterol, height, weight, and smoking status (15). 

Surveys at Rocky Boy’s were conducted before and immediately after the campaign and included standardized questions about heart attack warning signs and symptoms, immediate responses if witnessing an event, and respondents’ recall of advertisements regarding warning signs and symptoms. Unaided questions assessed respondents’ knowledge of heart attack warning signs and actions to take if witnessing a potential heart attack. Respondents were prompted to name up to 5 heart attack warning signs (“From what you may have heard or read, please tell me 5 warning signs or symptoms people may experience when having a heart attack”) and were asked 2 unaided questions adapted to identify what 2 actions they would take if they witnessed someone having a heart attack (“If you thought someone was having a heart attack, what is the first thing you would do?” and “What is the second thing you would do if you thought someone was having a heart attack?”) Finally, respondents were asked to recall whether in the previous 3 months they remembered seeing or hearing any radio or newsprint advertisements about heart attack warning signs and the importance of calling 9-1-1 immediately. Consistent with recommendations from national organizations, the following were the heart attack warning signs and symptoms emphasized during the public education campaign: chest pressure, pain, or discomfort; pain in arms, neck, jaw, or stomach; sweating or nausea; shortness of breath; fatigue; and anxiety (16).

At Fort Belknap, 3 separate telephone surveys were conducted to evaluate the stroke awareness campaign: baseline, postcampaign, and after the 4-month maintenance phase was completed. The stroke survey for AI residents of the Fort Belknap reservation included standardized questions about identifying stroke signs and symptoms, immediate response if witnessing a person having a stroke or if they themselves were having a stroke, and media recall. To assess respondents’ knowledge of stroke warning signs, unaided questions adapted from previous surveys were used (17). Respondents were prompted to name up to 3 stroke warning signs or symptoms with the following question, “From what you may have heard or read, please tell me 3 warning signs or symptoms people may experience when having a stroke.” To assess immediate response to witnessing or experiencing a stroke, respondents were asked the following 4 unaided questions adapted from previous surveys: 1) “If you thought someone was having a stroke, what is the first thing you would do?”, 2) “If you experienced sudden difficulty speaking, reading, or understanding that would not go away, what is the first thing you would do?”, 3) “If you experienced sudden numbness, tingling, or dead sensation on 1 side of your body that would not go away, what is the first thing you would do?”, and 4) “If you experienced sudden weakness or paralysis on 1 side of your body that would not go away, what is the first thing you would do?” Finally, to assess media recall, respondents were asked at baseline, postcampaign, and postmaintenance if in the previous 3 months they remembered seeing or hearing any radio or newsprint advertisements about stroke warning signs and the importance of calling 9-1-1. Consistent with current recommendations from a national organization, the sudden onset of the following symptoms were considered to be a warning sign for stroke: dizziness, difficulty understanding or slurred speech, severe headache, problems with vision, weakness on 1 or both sides of body or face, numbness on 1 or both sides of body or face, trouble walking, loss of balance, or lack of coordination (18). These were the signs and symptoms emphasized during the stroke public education campaign.

Data analyses were completed using SPSS version 17.0 software (SPSS Inc, Chicago, Illinois). We used χ^2^ tests to evaluate the effect of the heart attack and stroke campaigns by comparing differences from baseline to postcampaign in respondents’ knowledge of 3 or more heart attack warning signs or 2 or more stroke warning signs, recall of media messaging, and the importance of calling 9-1-1. A *P* value of <.05 was considered significant. 

## Results

### Rocky Boy’s heart attack awareness campaign

A total of 659 interviews were completed from Rocky Boy’s reservation (n = 292, baseline survey; n = 367, postcampaign survey). At baseline and postcampaign, no significant differences were found in the characteristics of the participants (Table 1). Overall, approximately one-quarter of the respondents reported a history of diabetes, 45% hypertension, and 50% high cholesterol; 56% were smokers. Seventy-eight percent were overweight or obese. Eleven percent had a history of heart attack; 2% reported having a previous stroke; and 8% reported having had an angioplasty or cardiac bypass surgery (data not shown).

**Table 1:** Demographic Characteristics and Cardiovascular Risk Factors Among American Indians Living on or Near the Rocky Boy’s or Fort Belknap Reservations, at Baseline, Postcampaign, and Postmaintenance, Montana, 2008–2010

Characteristic/Risk Factor	Rocky Boy’s Reservation, Heart Attack			Fort Belknap Reservation, Stroke			
	Baseline (n = 292)	Postcampaign (n = 367)	*P* Value	Baseline (n = 354)	Postcampaign (n = 400)	Postmaintenance (n = 400)	*P* Value
**Mean age (SD), y**	50.0 (13.3)	49.8 (12.5)	.40	51.6 (13.6)	53.5 (13.7)	54.4 (14.1)	.02
**Female, n (%)**	175 (60.0)	232 (63.2)	.39	204 (57.6)	245 (61.3)	247 (61.8)	.46
**Education, n (%)**							
12th grade or less	132 (45.4)	158 (43.2)	.72	133 (37.8)	163 (40.9)	156 (39.1)	.85
Some college	90 (30.9)	124 (33.9)		127 (36.1)	136 (34.1)	148 (37.1)	
≥4 years of college	69 (23.7)	84 (23.0)		92 (26.1)	100 (25.1)	95 (23.8)	
**Health care coverage, n (%)**	263 (90.4)	326 (89.1)	.59	325 (92.1)	366 (91.5)	390 (97.5)	.001
**Diabetes, n (%)**	74 (25.3)	89 (24.3)	.75	94 (26.6)	91 (22.8)	110 (27.5)	.27
**Hypertension, n (%)**	136 (46.7)	161 (43.9)	.46	187 (53.0)	218 (54.5)	232 (58.0)	.36
**High cholesterol, n (%)**	109 (53.4)	129 (46.1)	.11	117 (44.8)	138 (42.6)	133 (42.6)	.83
**Smoker, n (%)**	174 (59.8)	191 (52.3)	.06	155 (43.8)	167 (41.8)	156 (39.0)	.41
**Body mass index, kg/m^2^, n (%)**							
<25.0 (Normal weight)	64 (23.0)	76 (21.2)	.22	55 (16.5)	72 (18.9)	89 (23.3)	.11
25.0–29.9 (Overweight)	82 (29.5)	129 (36.0)		113 (33.9)	141 (37.1)	119 (31.2)	
≥30.0 (Obese)	132 (47.5)	153 (42.7)		165 (49.5)	167 (43.9)	174 (45.5)	

The proportion of respondents who correctly reported 3 or more heart attack warning signs and symptoms increased significantly from 35% at baseline to 47% postcampaign (Table 2). Three-quarters of respondents at baseline recognized the need to call 9-1-1 for heart attack, and the percentage remained unchanged postcampaign (data not shown).

**Table 2:** Knowledge of Correct Heart Attack or Stroke Warning Signs Among American Indians Living on or Near Rocky Boy’s or Fort Belknap Reservation, Respectively, at Baseline, Postcampaign, and Postmaintenance, Montana, 2008–2010

No. of Warning Signs	Rocky Boy’s Reservation, Heart Attack			Fort Belknap Reservation, Stroke			
	Baseline, n (%) (N = 292)	Postcampaign, n (%) (N = 367)	*P* Value	Baseline, n (%) (N = 354)	Postcampaign, n (%) (N = 400)	Postmaintenance, n (%) (N = 400)	*P* Value
0	29 (9.9)	26 (7.1)	.01	58 (16.4)	33 (8.3)	42 (10.5)	<.001
1	55 (18.8)	61 (16.6)		77 (21.8)	66 (16.5)	67 (16.8)	
2	105 (36.0)	109 (29.7)		132 (37.3)	154 (38.5)	133 (33.3)	
3	71 (24.3)	92 (25.1)		87 (24.6)	147 (36.8)	158 (39.5)	
4	23 (7.9)	56 (15.3)		NA	NA	NA	NA
5	9 (3.1)	23 (6.3)		NA	NA	NA	NA
≥2	NA	NA	NA	219 (61.9)	301 (75.3)^a^	291 (72.8)^b^	<.001
≥3	103 (35.3)	171 (46.6)	.003	NA	NA	NA	NA

a Comparison between baseline and postcampaign.

b Comparison between baseline and postmaintenance.

**Figure 1:**
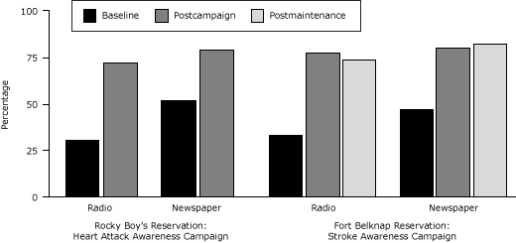
Survey respondents’ recall of radio and newspaper advertisements at baseline and postcampaign for Rocky Boy’s Reservation, and at baseline, postcampaign, and postmaintenance for Fort Belknap Reservation, Montana, 2008–2010. **Table:** 

Among Rocky Boy’s survey respondents, recall of media messaging for the radio advertising more than doubled from 31% at baseline to 72% postcampaign (Figure 1). Overall, recall rates for the print campaign, which included newspaper advertisements, print inserts, and magazine advertisements, also increased from 52% at baseline to 79% postcampaign.

Medium	Rocky Boy’s Reservation, Heart Attack, %		*P* Value	Fort Belknap Reservation, Stroke, %			*P* Value
	Baseline	Postcampaign		Baseline	Postcampaign	Postmaintenance	
Radio	31	72	<.05	33	77	74	<.001
Newspaper	52	79		47	80	82

### Fort Belknap stroke awareness campaign

A total of 1,154 telephone interviews were conducted at Fort Belknap (n = 354, baseline; n = 400, postcampaign; n = 400, postmaintenance). Approximately 60% of survey respondents were female for each of the surveys, and the average age of respondents ranged from 51.6 years to 54.4 years; respondents to the postmaintenance survey were slightly older than respondents to the baseline survey (Table 1). Approximately 92% reported having health care coverage in the baseline and postcampaign surveys; however, the percentage increased significantly to 97% in the postmaintenance survey. Overall, approximately one-quarter of participants reported a history of diabetes, 55% hypertension, 43% high cholesterol, 41% current smoking, and 78% were overweight or obese. No significant differences in reported history of these cardiovascular disease risk factors were found between baseline, postcampaign, and postmaintenance. A total of 8% of respondents reported a history of heart attack, and 3% reported a history of stroke (data not shown).

**Figure 2:**
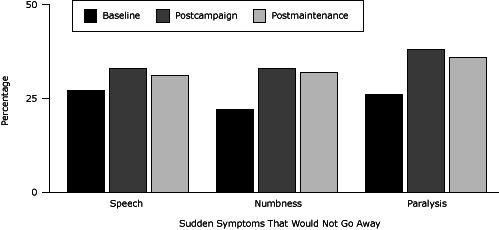
Survey respondents who would call 9-1-1 if They Experienced Stroke Symptoms, at baseline, postcampaign, and postmaintenance, Fort Belknap Reservation, Montana, 2008–2010. **Table:** 

The proportion of respondents correctly identifying 2 or more stroke warning signs and symptoms increased significantly from 62% at baseline to 75% immediately following the high-intensity campaign, and this recognition remained high at 73% after the maintenance campaign (Table 2). At baseline, two-thirds of respondents recognized the need to call 9-1-1 if witnessing someone having a stroke, and the percentage remained unchanged after the high-intensity campaign and after the maintenance campaign (data not shown). After the high-intensity campaign, significant increases were found in intent to call 9-1-1 if the person experienced numbness (from 22% to 33%) or paralysis (from 26% to 38%) that did not go away; the increase in intent to call 9-1-1 persisted after the maintenance period (Figure 2).

Period	Experience Sudden Symptoms That Would Not Go Away, %					
	Speech	*P* Value	Numbness	*P* Value	Paralysis	*P* Value
Baseline	27	.16	22	.004	26	.002
Postcampaign	33		33		38	
Postmaintenance	31		32		36

At baseline, 33% reported hearing radio advertisements (Figure 1). The percentage increased significantly to 77% at postcampaign and declined slightly to 74% at postmaintenance. Recall of newspaper exposure also increased significantly from 47% at baseline to 80% at postcampaign and 82% postmaintenance.

## Discussion

Awareness of the warning signs of heart attack and stroke increased in Montana AI communities after public education campaigns developed in cooperation with tribal partners. Direct mailings, newspaper advertisements, posters, and print inserts featuring recognizable people were used over several months in each community. The survey responses indicated these simple methods could be used successfully to reach rural reservation residents. Road signs on the main highway from town and advertising at local movie theaters in nearby towns were combined with advertisements posted on YouTube. It was not feasible to use television advertisements because in rural areas most residents received television from distant cities via satellite.

The lack of change in intent to call 9-1-1 found in the surveys was puzzling. At baseline and postcampaign, respondents were much less likely to say they would call 9-1-1 than were respondents from other similar surveys in Montana. More than 80% of state employees reported that they would call 9-1-1 for stroke or heart attack (19). Three-quarters of those surveyed in Missoula County responded that they would call 9-1-1 if they witnessed a stroke (20). The lack of change in intent to call 9-1-1 may be due in part to barriers particular to rural emergency medical services (EMS). Volunteer first responders may not be immediately available for dispatch at all hours, and they may have to travel long distances to reach people having a heart attack or stroke. Respondents identified long distance from the health care facility as a key barrier to calling 9-1-1, and some reported it would be much faster if they went directly to the hospital themselves for treatment rather than waiting for EMS to arrive. In addition, community members participating in the focus groups were not confident that EMS would be able or available to respond after hours.

Our study has limitations. We used telephone surveys, so households without telephones were not included. Second, we used self-reported data, which are subject to recall bias. Third, respondents were asked “unaided” questions to assess their knowledge of the warning signs and intention to call 9-1-1, so the awareness of warning signs may have been underestimated. Finally, because of the size of the 2 communities, individuals may have been interviewed more than once and may have become familiar with the questions and may have been eager to provide a correct response.

Despite the limitations of the survey, the findings are encouraging and relevant. In Montana, deaths from heart disease and stroke declined in the white population between 1991 and 1995 compared with the period from 1996 to 2000, but rates changed very little among Montana’s AI population during the same period (12). The prevalence of cardiovascular risk factors was high in AI communities in Montana and increased between 1999 and 2003 (21). The telephone surveys associated with the campaigns confirmed the presence of high-risk populations on each reservation. By working closely with tribal health departments, it was possible to develop and implement public education campaigns that many community members noticed. There were significant increases in campaign message recall from baseline to postcampaign, despite the fact that no television advertisements were used.

Challenges remain with measuring the sense of urgency to seek treatment in remote settings. Intent to call 9-1-1 may not have the same importance among residents of rural areas compared with those of urban areas. More research and a better understanding of the logistics of accessing EMS in rural settings are needed to understand this issue better. Nonetheless, the campaigns had an effect on the knowledge of cardiovascular warning signs and symptoms in a high-risk population of adults aged 30 years or older and also may have influenced younger people. Carefully designed, culturally specific campaigns may help eliminate disparities in the recognition of heart attack and stroke warning signs in AI communities.

## Figures and Tables

**Table:** 

**1. The activity supported the learning objectives.**				
**Strongly Disagree**				**Strongly Agree**
1	2	3	4	5
**2. The material was organized clearly for learning to occur.**				
**Strongly Disagree**				**Strongly Agree**
1	2	3	4	5
**3. The content learned from this activity will impact my practice.**				
**Strongly Disagree**				**Strongly Agree**
1	2	3	4	5
**4. The activity was presented objectively and free of commercial bias.**				
**Strongly Disagree**				**Strongly Agree**
1	2	3	4	5

## Post-Test Information

To obtain credit, you should first read the journal article. After reading the article, you should be able to answer the following, related, multiple-choice questions. To complete the questions (with a minimum 70% passing score) and earn continuing medical education (CME) credit, please go to http://www.medscape.org/journal/pcd. Credit cannot be obtained for tests completed on paper, although you may use the worksheet below to keep a record of your answers. You must be a registered user on Medscape.org. If you are not registered on Medscape.org, please click on the "Register" link on the right hand side of the website to register. Only one answer is correct for each question. Once you successfully answer all post-test questions you will be able to view and/or print your certificate. For questions regarding the content of this activity, contact the accredited provider, CME@medscape.net. For technical assistance, contact CME@webmd.net. American Medical Association's Physician's Recognition Award (AMA PRA) credits are accepted in the US as evidence of participation in CME activities. For further information on this award, please refer to http://www.ama-assn.org/ama/pub/about-ama/awards/ama-physicians-recognition-award.page. The AMA has determined that physicians not licensed in the US who participate in this CME activity are eligible for **AMA PRA Category 1 Credits™**. Through agreements that the AMA has made with agencies in some countries, AMA PRA credit may be acceptable as evidence of participation in CME activities. If you are not licensed in the US, please complete the questions online, print the AMA PRA CME credit certificate and present it to your national medical association for review.

## Post-Test Questions

### Article Title: Cooperative Strategies to Develop Effective Stroke and Heart Attack Awareness Messages in Rural American Indian Communities, 2009–2010

**CME Questions**

1. You are a primary care physician working in a community clinic on an Indian reservation. You just received a message from the daughter of one of your patients that your patient is in the hospital for evaluation and treatment of heart attack. The patient is 70 years old with a history of diabetes mellitus and hypertension. Compared with the total US population, heart attack and stroke mortality rates in American Indians are:
  - Lower in both heart attack and stroke
  - Lower in heart attack and higher in stroke
  - Higher in heart attack and lower in stroke
  - Higher in both heart attack and stroke
2. You visit the patient in the hospital. Upon speaking with her and the daughter, you learn that the patient has been experiencing intermittent chest pressure and fatigue for the past 2 days. She believed it was just exhaustion from working in the garden in the past week. She and her daughter had not been concerned about a heart attack. This has not been the first time you have encountered patients within this community who were unaware of signs and symptoms of a heart attack. According to this study by Oser and colleagues, to increase awareness of warning signs of heart attack and stroke in rural AI communities, which method(s) for public education campaigning should be utilized?
  - Direct mailings
  - Road signs on the main highway
  - Television advertisements
  - A and B only
  - All of the above
3. According to this study by Oser and colleagues, public education campaigns regarding heart attack and stroke awareness led to which of the following:
  - Knowledge of 3 or more heart attack warning signs and symptoms increased significantly from 35% at baseline to 47% post campaign within 1 reservation
  - Among the Rocky Boy’s heart attack awareness campaign, recall rates for the print campaign decreased from baseline to post campaign while recall of media messaging increased from baseline to post campaign
  - Recognition of 2 or more stroke signs and symptoms increased from 62% to 70% post campaign within the second reservation
  - In the Fort Belknap stroke awareness campaign, there was no change from recognition of symptoms from baseline to postmaintenance campaign
4. In regards to need to seek treatment, this study demonstrated that the intent to call 9-1-1 after both heart attack and stroke awareness campaigns were:
  - Decreased in heart attack but increased in stroke
  - Increased in heart attack but decreased in stroke
  - Increased in both heart attack and stroke
  - Unchanged in both heart attack and stroke

**Evaluation**
